# A“Proteoglycan Targeting Strategy” for the Scintigraphic Imaging and Monitoring of the Swarm Rat Chondrosarcoma Orthotopic Model

**DOI:** 10.1155/2011/691608

**Published:** 2011-02-06

**Authors:** Caroline Peyrode, François Gouin, Aurélien Vidal, Philippe Auzeloux, Sophie Besse, Marie-Mélanie Dauplat, Serge Askienazy, Dominique Heymann, Jean-Michel Chezal, Françoise Redini, Elisabeth Miot-Noirault

**Affiliations:** ^1^INSERM (UMR 990) Université d'Auvergne, rue Montalembert, BP 184, 63005 Clermont-Ferrand Cédex, France; ^2^INSERM (UMR S957), Université de Nantes, 44035 Nantes, France; ^3^Service d'Anatomo-Pathologie, CLCC Jean Perrin, 63001 Clermont-Ferrand, France; ^4^Cyclopharma Laboratoires, 63360 Saint-Beauzire, France

## Abstract

Our lab developed ^99m^Tc-NTP 15-5 radiotracer as targeting proteoglycans (PGs) for the scintigraphic imaging of joint. This paper reports preclinical results of ^99m^Tc-NTP 15-5 imaging of an orthotopic model of Swarm rat chondrosarcoma (SRC). ^99m^Tc-NTP 15-5 imaging of SRC-bearing and sham-operated animals was performed and quantified at regular intervals after surgery and compared to bone scintigraphy and tumoural volume. Tumours were characterized by histology and PG assay. SRC exhibited a significant ^99m^Tc-NTP 15-5 uptake at very early stage after implant (with tumour/muscle ratio of 1.61 ± 0.14), whereas no measurable tumour was evidenced. As tumour grew, mean tumour/muscle ratio was increased by 2.4, between the early and late stage of pathology. Bone scintigraphy failed to image chondrosarcoma, even at the later stage of study. ^99m^Tc-NTP 15-5 imaging provided a suitable set of quantitative criteria for the *in vivo* characterization of chondrosarcoma behaviour in bone environment, useful for achieving a greater understanding of the pathology.

## 1. Introduction

Our laboratory has developed the ^99m^Tc-N-[triethylammonium]-3-propyl-[15]ane-N5 (^99m^Tc-NTP 15-5) radiotracer able to link proteoglycans (PGs) of cartilage, which was demonstrated to allow scintigraphic imaging of joint [1–6]. PG appear also as key partners in bone cell biology, and might represent interesting targets for the assessment of malignant pathological processes [7]. Chondrosarcoma represent 25% of all bone sarcoma and are characterized by the formation of a cartilage like extracellular matrix [8–10]. To the orthopedic oncologists, it is essential to identify the lesions that are thought to constitute the “cartilage tumour group”, through the presence of identifiable elements that resemble the cells and matrix of cartilage. For such analysis, the three-grade histopathologic classification of Evans is based on cell type/differentiation, matrix formation and architecture, and is at present considered useful for the prediction of clinical behaviour [11–13]. To date, many questions still remain unanswered and there is an urgent need for markers of biologic phenotypic features, that could be determined in a noninvasive manner, and could be used as objective criteria of grading, followup, response to therapy and detection of recurrence. CT and MRI define morphology, but are limited in distinguished postoperative residual and postchemotherapeutic lesions due to altered tissue planes, edema and fibrosis. Contrast-enhanced MRI, while providing data for local staging and extraosseous involvment, appears also limiting in determining biologic behaviour of lesions [14–16]. SPECT by the use of highly specific radiolabelled probes, allows a noninvasive and quantitative assessment of many biochemical pathways *in vivo* [17]. In such context, scintigraphic imaging with ^99m^Tc-NTP 15-5 would be a powerful tool for a direct *in vivo* quantitative evaluation of chondrosarcoma at the PG level, allowing therefore the detection of changes in relation to pathological processes and/or response to therapies. We therefore initiated preclinical studies aiming at determining whether ^99m^Tc-NTP 15-5 imaging could be useful for the evaluation of the tumoural pathology of cartilage *in vivo*. In the present study, the ^99m^Tc-NTP 15-5 imaging was characterized in the Swarm rat chondrosarcoma implanted in paratibial location.

## 2. Materials and Methods

### 2.1. Animals

Male Sprague Dawley (Charles River, France) were included in this study. They were handled and cared for in accordance with the guidelines for the Care and Use of Laboratory Animals (National Research Council, 1996) and European Directive 86/809/EEC. They were maintained at 21°C with a 12 h/12 h light/dark cycle. Protocols were performed under the authorisation of the French Direction of Veterinary Services (Authorisation N° C63-113-10) and conducted by authorized investigators in accordance with the institution's recommendations for the use of laboratory animals.

### 2.2. Chondrosarcoma Model

The Swarm rat chondrosarcoma (SRC) line was a generous gift from Dr. P. A. Guerne (Geneva, Switzerland) as tissue fragments that were frozen until use. 

For tumoural implant, the rats were anaesthetized by inhalation of a combination isoflurane (Abbott, France)/air (1.5%, 1 L/min) associated with an intramuscular injection of 100 mg/kg ketamine (Imalgene, Rhone Merieux, France).

Allograft transplantation of a tumour fragment (10 animals) was performed on the right paw, the other paw being used as the controlateral reference, as previously published [18].

 Using a lateral approach, the cortical surface of the diaphysis was scarified laterally on 10 mm, a 10 mm^3^ fragment of SRC was placed contiguous to the scarified surface, and the muscular and cutaneous wounds were sutured. The same procedure was performed for the controlateral paw, but no tumour fragment was implanted. Tumours appeared at the graft site 7–11 days later.

### 2.3. Establishment of the Sham Model

Five animals underwent sham surgery, in which the right paw was submitted to the same surgery procedure as SRC animals, but no tumour fragment was implanted.

### 2.4. Acquisitions for ^99m^Tc-NTP 15-5 Imaging

Scintigraphic *in vivo* imaging was performed using a small-animal *γ* camera (CsI(Na) crystal) equipped with a 1.3/0.2/35 parallel-hole collimator (hole diameter/septum thickness/height in mm) (Gammaimager, Biospace, France). The energy resolution and intrinsic planar resolution of the system are given as 11% at 140 keV and <2 mm full width at half maximum (FWHM), respectively. 

**Figure 1:**
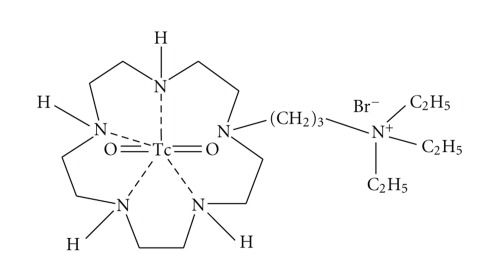
Chemical structure of ^99m^Tc-N-[triethylammonium]-3-propyl-[15]ane-N5 (^99m^Tc-NTP 15-5).

The radiotracer ^99m^Tc-NTP 15-5 (Figure 1) radiotracer was prepared and radiolabelled as previously published [1–6], and administered by IV route to vigil animals (25 Mbq/animal) using dedicated contention box. 

**Figure 2:**
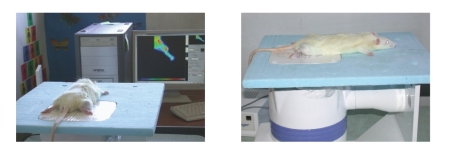
*In vivo* scintigraphic acquisition of chondrosarcoma bearing rats using a small animal dedicated gamma camera.

First of all, a 10-min planar acquisition (with a 15% window centered on the 140 keV photopeak of ^99m^Tc) was performed on 3 animals with a well-established tumour (volume = 949.35 ± 223 mm^3^) at several intervals (30, 60, 90 and 120 mins) to determine kinetics of tracer accumulation within chondrosarcoma, respectively, to articular cartilage and muscle. For acquisition, each posterior paw of animals was positioned over the collimator of the camera as illustrated in Figure 2. Quantitative analysis of scintigraphic scans was performed using the GAMMAVISION+ software (Biospace, France). Regions of interest (ROIs) were delineated over tumour, femorotibial cartilage and muscle patterns. For each animal and each ROI, average count in cpm per pixel was obtained. Two semiquantitative parameters were determined:

(1) $$\begin{array}{c} \frac{\text{T}}{\text{M}}=\frac{\text{average}\;\text{count}\;\text{in}\;\text{tumour}}{\text{average}\;\text{count}\;\text{in}\;\text{muscle}},\\ \frac{\text{T}}{\text{C}}=\frac{\text{average}\;\text{count}\;\text{in}\;\text{tumour}}{\text{average}\;\text{count}\;\text{in}\;\text{femorotibial}\;\text{cartilage}}. \end{array}$$

Data were expressed as mean ± standard deviation.

 A serial imaging was then performed on primary chondrosarcoma-bearing rats (*n* = 10) and sham-operated animals (*n* = 5) at regular intervals after surgery (from day 4 to day 35), with 10-min planar acquisition being started 30 mins after iv administration of ^99m^Tc-NTP 15-5. Scintigrams were considered positive when tracer uptake areas corresponded to sites of implant. All the positive scans were evaluated using the ROI method as described above, with T/C and T/M parameters being determined for each animal at each time point, and data expressed as mean ± standard deviation. Semi-quantitative parameters determined at day 4 were used as the “threshold reference value” and were compared to the mean values determined at each time point of study (paired 2-sided Student *t*-test with a level of significance set at *P* < .05).

### 2.5. Acquisitions for ^99m^Tc-HMDP Bone Imaging

Primary chondrosarcoma-bearing rats were also submitted to bone scintigraphies at day 12, day 38, and day 48 after implant. For each paw, “delayed images” (10-minute duration) were acquired 2 hours post injection of ^99m^Tc-HMDP radiotracer (Osteocys, IBA, France; 30 MBq/animal). 

Bone scintigrams were considered positive when tracer uptake areas corresponded to sites of implant. 

### 2.6. Tumour Growth Assessment

The tumour volume was determined from the measurement of 2 perpendicular diameters using a calliper. Tumour volumes (*V*) were calculated according to the following formula:

(2) $$V\left({\text{mm}}^{3}\right)=0.5\times L\times {\left(S\right)}^{2},$$

where *L* and *S* are, respectively, the largest and smallest perpendicular tumour diameters in mm [19]. 

### 2.7. Histology of SRC Tissue

At the end of the study, three chondrosarcoma-bearing rats were killed for histological characterization of the tumours. The femora and tumour of each rat were removed and fixed at 4°C for 48 hours in formol buffer (pH 7.4). The femora where cut longitudinally or transversally. Decalcified femoral fragments were embedded in paraffin and 5 *μ*m sections were mounted on glass slides for staining with Hematoxylin-Eosin-Safran (HES) and Alcian blue.

### 2.8. PG Content of SRC Tissue

At the end of the study, SRC were removed (*n* = 3 animals), dissected and digested for 24 h at 60°C with EDTA-phosphate buffer solution containing Papaine 0.60 mg/mL (Sigma-Aldrich, France) and DL-dithiothreitol (DTT) 0.25 mg/mL (Sigma-Aldrich, France). Digests were then assessed for PG content using the dimethylene blue protocol [20]. 

 PG content of SRC tissue was compared to PG content of mouse B16F0 melanoma as negative control: B16F0 melanoma were removed from bearing mice (*n* = 3) (stage day 15 after subcutaneous inoculation of 300000 B16F0 cells), and submitted to the same procedure of extraction and digestion as SRC tumours.

## 3. Results

### 3.1. SRC Characterisation: Tumoural Growth, Histology and PG Assay

**Figure 3:**
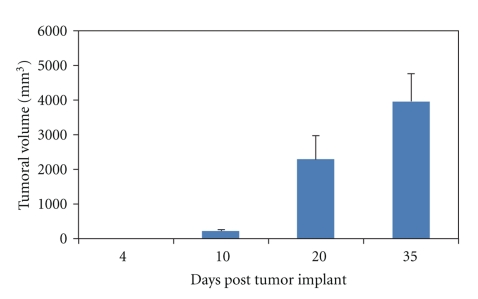
Tumour volume growth for Swarm rat chondrosarcoma (SRC) implanted in paratibial location. Mean values + standard deviation are presented at each time point.

**Figure 4:**
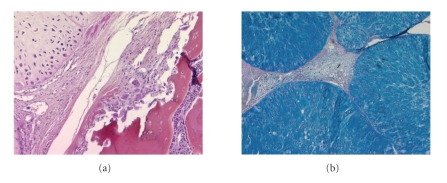
Histomorphological features of the SRC model at study end showing: (a) a tumoural tissue with hypercellularity, and bone osteolysis (x10); (b) a high density of blue Alcian stained areas as a reflect of proteoglycan content (x5).

Tumour volumes were followed for 35 days after primary implant (Figure 3). All the animals developed a tumour that became palpable from day 10. Histological examination (Figure 4(a)) at study end evidenced a chondroid tumoural tissue, poorly vascularized, lobular in organization with lobules containing chondroid stroma and delimited by fine fibrous septa. Hypercellularity was also observed. At this later stage of pathology, an extensive invasion of bone and surrounding tissues was present, as well as osteolysis. 

The overall presence of PG was first investigated by Alcian blue staining: as shown in Figure 4(b), a high density of stained areas were observed in SRC tissue, revealing the presence of PG in this tumour.

**Figure 5:**
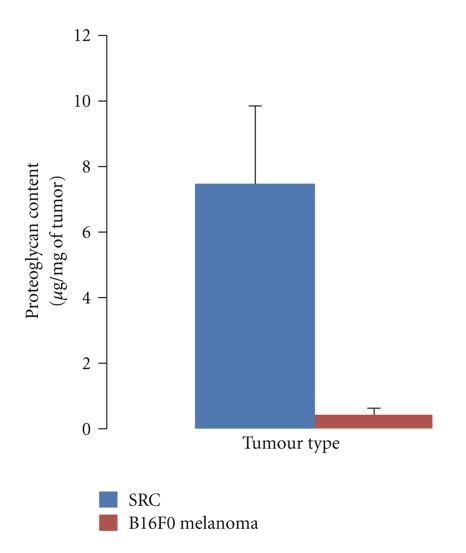
PG content of SRC tissue, respectively, to murine melanoma tumour (as negative control); Mean values + standard deviation are presented.

PG content of SRC tumour tissue was also assessed by biochemical dosage. PG content of SRC tissue was compared with PG of murine B16F0 melanoma tumour as negative control: As shown in Figure 5, a high PG content was observed in SRC tissue (7.46 ± 2.41 *μ*g of PG/mg of tissue), respectively, to melanoma (0.45 ± 0.16 *μ*g of PG/mg of tissue).

### 3.2. ^99m^Tc-NTP 15-5 Distribution in SRC Tissue, Respectively, to Muscle and Cartilage

**Figure 6:**
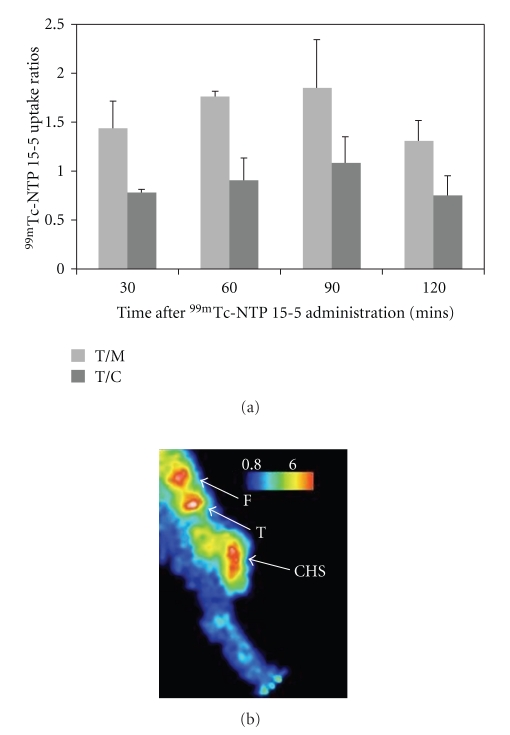
^99m^Tc-NTP 15-5 *in vivo* distribution in chondrosarcoma; (a) ^99m^Tc-NTP 15-5 uptake ratios as a function of delay between iv administration of tracer and acquisition; (b) Scintigraphic image obtained for a representative animal, for a delay of 30 min between injection and acquisition. Abbreviations: F: femoral condyle; T: tibial plateau; CHS: chondrosarcoma.

^99m^Tc-NTP 15-5 distribution in well-established primary chondrosarcomas was characterized at 30, 60, 90 and 120 mins after ^99m^Tc-NTP 15-5 administration, on the basis of tumour/muscle (T/M) and tumour/cartilage (T/C) ratios. As shown in Figure 6(a), ^99m^Tc-NTP 15-5 rapidly accumulated within SRC tissue, with T/M and T/C ratios being 1.44 ± 0.27 and 0.78 ± 0.03 from 30 mins post injection. Radiotracer accumulation within tumoural tissue was observed to be as high as in cartilage tissue from 60 mins pi, with T/C value of 0.90 ± 0.23. No significant changes were observed in T/M and T/C ratios from 60 to 120 mins pi.

This high and selective accumulation of ^99m^Tc-NTP 15-5 within tumour was at the origin of the highly contrasted chondrosarcoma imaging *in vivo*, as shown in (Figure 6(b)) for a representative animal.

### 3.3. Diagnosis Capability of ^99m^Tc-NTP 15-5 Imaging, Respectively, to ^99m^Tc-HMDP Bone Scintigraphy and Calliper Measurement

All the implanted animals were positive for chondrosarcoma at necropsy.

**Figure 7:**
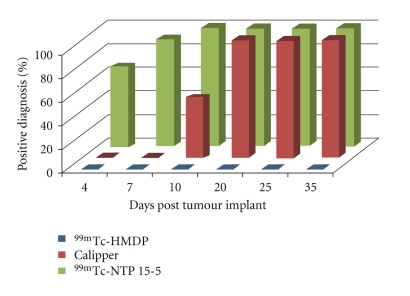
Disease incidence at each time point of study, according to ^99m^Tc-HMDP scintigraphy, ^99m^Tc-NTP 15-5 imaging, and calliper measurement.

Figure 7 shows the disease incidence according to ^99m^Tc-HMDP scintigraphy, ^99m^Tc-NTP 15-5 imaging, and calliper measurement during the whole study of monitoring. 

A palpable and measurable tumour was observed from day 10 after implant for 50% of the animals. From day 20, 100% of the animals evidenced measurable tumours. 

^99m^Tc-NTP 15-5 scintigraphy was positive for 66.66% of the animals as early as day 4 post implant, and for 100% of the animals from day 10. 

^99m^Tc-HMDP bone scintigraphy was negative for SRC imaging during the whole duration of study, and even later (48 days post implant).

### 3.4. Monitoring of SRC Growth In Vivo Using ^99m^Tc-NTP 15-5 Imaging

**Figure 8:**
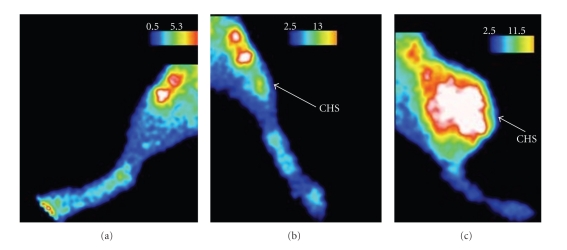
^99m^Tc-NTP 15-5 longitudinal *in vivo* examination of a representative SRC-bearing rat; (a) *in vivo* scintigraphic image of the contralateral paw at day 4; (b) *in vivo* scintigraphic image of the tumour-bearing paw at day 4; (c) *in vivo* scintigraphic image of the tumour bearing paw of the same animal at day 35. A clear accumulation of radioactivity within tumoural tissue was observed as early as day 4 after implant (Image b, arrow); abbreviations: CHS = chondrosarcoma.

As shown in Figure 8 for a representative animal, ^99m^Tc-NTP 15-5 radiotracer was observed to accumulate within tumoural tissue, respectively, to the contralateral paw (Figure 8(a)), as early as day 4 after implant: scintigram of the tumour bearing paw (Figure 8(b)) evidenced (i) uptake areas in femorotibial articular cartilage, with the tibial plateau uptake clearly distinguished from the femoral condyle uptake, (ii) accumulation at the site of implant. ^99m^Tc-NTP 15-5 examination of the same animal at late stage of pathology (day 35, Figure 8(c)) evidenced that radiotracer accumulation within the tumour-bearing paw was highly increased, respectively, to day 4.

**Figure 9:**
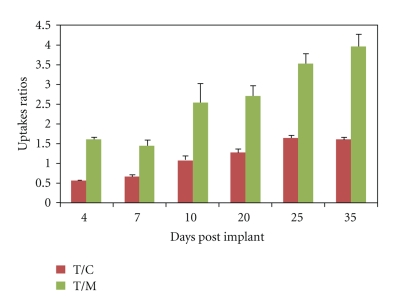
Quantitative analysis of ^99m^Tc-NTP 15-5 accumulation in chondrosarcoma against time after implant. Results are expressed as Tumour/Muscle and Tumour/Cartilage uptake ratios (T/M and T/C, resp.). Mean values + standard deviation are presented at each time point.

^99m^Tc-NTP 15-5 accumulation was quantitatively assessed *in vivo* in the tumour, respectively, to muscle and cartilage (as T/M and T/C uptake ratios, resp.) as a function of time after implant (Figure 9): As early as day 4 post implant, the mean T/M and T/C values were 1.61 ± 0.14 and 0.57 ± 0.06, respectively. From day 10, tumoural uptake of ^99m^Tc-NTP 15-5 was as high as cartilage uptake with T/C = 1.08 ± 0.09; T/M value was 2.55 ± 1.15.

At study ending (day 35), mean T/C and T/M values were 1.61 ± 0.06 and 3.97 ± 0.74, respectively.

### 3.5. Monitoring of Sham-Operated Animals Using ^99m^Tc-NTP 15-5 Imaging

Serial ^99m^Tc-NTP 15-5 imaging of sham-operated animals (*n* = 5) performed over 50 days after surgery did not evidence any accumulation of the tracer at site of surgery.

## 4. Discussion

This experimental study was conducted in the syngeneic model of SRC that has been the subject of extensive biochemical studies on extracellular matrix and chondrocyte metabolism [21, 22]. Histological characterization of SRC implanted in paratibial location, evidenced a chondroid tumoural tissue, poorly vascularized, lobular in organization with lobules containing chondroid stroma delimited by fine fibrous septa. In the present study PG were confirmed (by both alcian blue staining and biochemical assay) as being a major component of SRC tissue. The high concentration of PG in the tumour is at the origin of a high density of ECM strong negative charges that may interact with the positively charged quaternary ammonium moiety of the ^99m^Tc-NTP 15-5 radiotracer. The ability of ^99m^Tc-NTP 15-5 to image SRC tissue was therefore demonstrated from 30 mins post injection with T/M and T/C ratios being 1.44 ± 0.27 and 0.78 ± 0.03.

Interestingly, tracer accumulation was detected at early stage (day 4) and for about 70% of the animals investigated, whereas no palpable nor measurable tumour was evidenced. Such early accumulation raised the question of whether the imaging pattern represented an effective tumoural uptake or a surgery-induced inflammation. Negative ^99m^Tc-NTP 15-5 imaging observed for sham-operated animals definitely ruled out the hypothesis of inflammation contribution. 

^99m^Tc-NTP 15-5 accumulation within SRC was assessed at multiple time-points of tumoural growth using a semiquantitative method. As tumour developed over time, mean T/M and T/C values were observed to be increased by a factor of 2.4 and 2.8, respectively, between the early stage day 4 and the late stage day 35. Tumour to femorotibial cartilage uptake ratio (T/C parameter) is of particular importance for clinical application (in patients, tumour is located in the joint). From our results, the increase in T/C with pathology argued in favour of a high tumour/cartilage contrast which would expect diagnosis of tumour occurrence within the joint. 

Another interesting result of this study is that ^99m^Tc-HMDP bone scintigraphy commonly used in clinical practise failed to image tumoural tissue throughout the study, whereas ^99m^Tc-NTP 15-5 imaging was positive.

These preclinical results underlined the suitability and high sensitivity of ^99m^Tc-NTP 15-5 scintigraphic imaging for assessing *in vivo* the PG component of chondrosarcomas, providing therefore criteria for a quantitative and functional assessment of the tumoural pathology *in vivo*. 

Our results raised the question of whether ^99m^Tc-NTP 15-5 imaging patterns in benign cartilage tumours, (which are known to form a cartilaginous PG—rich matrix), would be the same as for malignant tumours. According to many histological studies, the neoplastic cells of benign cartilage tumours such as chondroblastoma did not show, at any point of their evolution, real cartilage matrix formation [13, 23]. As a consequence, a differential accumulation of ^99m^Tc-NTP 15-5 radiotracer could be expected. Since the biological nature of benign cartilage tumours is still debating, we will have to elucidate this aspect by performing a preclinical ^99m^Tc-NTP 15-5 imaging study of rodent models of enchondroma, (such as mutant mice for hedgehog signalling pathways or Ext1/Ext2 exostosin encoding gene) in parallel to the biological characterization of PG of tissue [23–25]. 

From a clinical point of view, the distinction between benign and low-grade malignant pathology, is known to be extremely difficult both at radiologically and histologically, and remains a challenge [9, 26]. All current imaging modalities have shown their limitations. Radiographs, CT, MRI and contrast enhanced MRI provide morphological data for local staging and extra-osseous involvement, but appeared limited in determining functional markers of postoperative recurrence, response or relapse to therapies [9, 27–29]. In such context, ^99m^Tc-NTP 15-5 radiotracer may serve as an adjunct to CT and MRI, by supplying quantitative data which would allow imaging and follow-up to be functionally rather than morphologically based. For functional imaging of cartilage neoplasms, radiotracers currently available provide indirect evaluations of the pathology, such as bone remodeling and inflammation features, but do not allow biological assessment of the tumour. Many tumour seeking agents such as ^201^Tl, ^99m^Tc-MIBI, ^99m^Tc-Tetrofosmin, ^99m^Tc-DMSA(V) and more recently ^18^F-FDG have been found useful in an initial diagnosis and grading, but they have also demonstrated their limitations for imaging chondrosarcoma with low cellularity and low vascularity [30–33]. ^99m^Tc-NTP 15-5 imaging would allow a regular *in vivo* follow up, in order to evidence any “upregulation” or “downregulation” synthesis of PG, as the reflect of potential malignant transformation, recurrence, response or relapse to therapies. We strongly believe in such potential application since ^99m^Tc-NTP 15-5 imaging recently evidenced in the SRC model a “downregulation synthesis” of PG as a result of anticancer treatment (unpublished data). 

Another potential application of ^99m^Tc-NTP 15-5 imaging in chondrosarcoma would be the early imaging of metastasis. As a consequence, the sensitivity of ^99m^Tc-NTP 15-5 imaging for the early evaluation of chondrosarcoma metastasis has to be determined in relevant preclinical animal models of chondrosarcoma with spontaneous metastasis [34, 35].

## 5. Conclusion

This preliminary work in the orthotopic SRC model underlined the suitability and high sensitivity of ^99m^Tc-NTP 15-5 radiotracer for imaging chondrosarcoma at the PG level. ^99m^Tc-NTP 15-5 imaging provided a suitable set of quantitative criteria for the *in vivo* characterization of chondrosarcoma behaviour in bone environment, which could be useful for achieving a greater understanding of the pathology.

In view of a potential clinical application, ^99m^Tc-NTP 15-5 imaging appears of interest for (i) the establishment of the cartilaginous nature of “musculoskeletal” tumours (ii) the *in vivo* assessment to PG changes associated to the evolution of pathological process, local recurrence, response or relapse to therapies. Additional preclinical studies are needed to investigate the potential of ^99m^Tc-NTP 15-5 imaging in the tumoral pathology of cartilage.
